# Innovative measurement, trade-off-synergy relationship and influencing factors for agricultural net carbon emissions and effective supply of agricultural products in China

**DOI:** 10.1016/j.heliyon.2024.e24621

**Published:** 2024-01-19

**Authors:** Lin Zhang, Chengzhi Cai

**Affiliations:** Economic Institute, Guizhou University of Finance and Economics, Guiyang, 550025, China

**Keywords:** Agricultural net carbon emissions, Effective supply of agricultural products, Trade-off-synergy relationship, Green technology innovation, SYS-GMM

## Abstract

Sensitive zone of global climate change has been formed in China, and it has become a hot topic how can agriculture ensure food security and the supply of important agricultural products while achieving the “Dual Carbon” goal in the country. Based on such background, this paper uses the IPCC carbon emission calculation method, environmental input-output model and economic-water-carbon coefficient method to measure agricultural net carbon emissions, adopts bivariate spatial auto-correlation analysis and SYS-GMM to explore separately the relationship between agricultural net carbon emissions and effective supply of agricultural products, as well as the carbon reduction effect, growth effect and reasonable range of green technology innovation. The results show that: (1) China's agricultural net carbon emissions reveal a spatial distribution of “higher in the east than in the west than in the center” and a temporal characteristic of increasing year by year; China's effective supply of agricultural products shows an increasing trend and a spatial distribution of “higher in the east than in the center than in the west” in 2006–2012 and “higher in the east than in the west than in the center” in 2013–2020. (2) In 2006, 2010, 2015 and 2020, the number of provinces that belong to low-low agglomeration trade-off zone, low-high agglomeration synergy zone, non-significant zone, high-low agglomeration non-trade-off-synergy zone and high-high agglomeration trade-off zone averagely accounted for 12.500 %, 30.000 %, 26.667 %, 9.167 % and 21.667 % of the totality, respectively. (3) The carbon reduction and production growth effects of green technology innovation both show an inverted “U-shape”, and green technology innovation is conducive to both reducing agricultural net carbon emissions and improving supply of agricultural products when it is within a reasonable range of greater than 0.930. (4) Green technology innovation not only has significant spatial and temporal heterogeneity impact, but also exhibits a differential effect on productive agricultural carbon emissions, agricultural trade carbon emissions, agricultural carbon sinks, total output of agricultural products and agricultural net imports in international trade. Therefore, it is proposed that China should establish and improve green technology innovation incubation platforms, guide all participants to ensure the investment and application of green technology products within a reasonable range, formulate and implement regional differential policies and plan in accordance with local conditions, drive ultimately coordinated promotion of agricultural carbon emission reduction and product supply guarantee and lay an important foundation for achieving high-quality economic development and efficient ecological protection.

## 1 Introduction

Since the United Nations established “2030 global sustainable development goals (SDGs)” and the Paris Conference published a new agreement to deal with global climate change, global warming issue has been increasingly concerned by many countries and regions like the United States, the European Union, the United Kingdom, Canada, Japan, Brazil and so on. Agriculture is a fundamental industry that supports national economy construction and development, provides an important material basis for human survival and ensures long-term peace and stability of the country. However, the concentration of greenhouse gas in atmosphere has risen rapidly because of modern agricultural activity patterns brought by farmland shrunk, technological progress, economic growth and other reasons. So, in simpler words, agricultural carbon emissions and their environmental improvement value cannot be ignored. Crippa et al. (2021) pointed out that food-system CO_2_ emissions alone exceeded 30 % of total greenhouse gases emissions in 2015, and the impact of global warming on agriculture was very clear and intuitive, especially the growth of crops under high temperature [1]. In August 2022, the “Blue Book of Climate Change in China 2022” released by the China Meteorological Administration to the public, showed that the trend of global warming was still continuing, and extreme weather and climate events such as high temperature and heavy precipitation in the country were becoming more and more intense, especially rare and continuous high temperature, heat wave and less rain weather in the Yangtze River basin, and where Sichuan, Chongqing, Anhui, Hubei, Hunan and Jiangxi altogether had a drought-affected farmland area of 8213.334 km^2^ in 2022, that is, a substantial losses of China's agricultural development has formed [2]. In October, Callahan and Mankin (2022) suggested that the extreme high temperature caused by climate change made about $16 trillion losses of global economy between 1992 and 2013, and found that serious heat damage in agriculture, health system and other fields has increased year by year [3]. It can be seen that in whether China or other countries and regions, a green and low-carbon transformation of agriculture is imminent. For China, the “13th Five-Year Controlling Greenhouse Gas Emissions”, the “14th Five-Year National Agricultural Green Development Plan”, the “Implementation Plan for Reducing Emissions and Fixing Carbon in Agriculture and Rural Areas” and other policy documents have injected new impetus into national agriculture to cope with climate change and new strength into green and low-carbon agricultural development from economic, social and environmental aspects. At the same time, these documents have expressed that sensitive zone of global climate change has been formed in China, and previous “one size fits all”, “carbon emissions rush” and other extreme carbon reduction actions to face global warming can no longer be taken. Next, it is important to standardize and scientifically reduce agricultural carbon emissions while making overall arrangements for food security and important agricultural products regulation and then realizing green and low-carbon transformation and sustainable development of agriculture. So, how do we stick to this important point when agricultural activities are still facing great pressure of carbon emission reduction? This paper will attempt to discuss the measurement, trade-off-synergy relationship and influencing factors step by step, and aims at making some complements to previous studies and providing corresponding information on and advice for solving practical problem and future research.

The United Nations Conference on Sustainable Development adopted “Transforming Our World: The 2030 Agenda for Sustainable Development” in 2015, which provided meaningful direction and guidance for all countries around the world to achieve 17 sustainable development goals, such as eradicating hunger, achieving food security and promoting sustainable agriculture, boosting sustainable, inclusive and sustainable economic growth, taking urgent action to address climate change and its impact, as well as protecting, restoring and promoting the sustainable use of resources and ecosystems, etc [4]. Since then, most countries in the world have begun to make continuous efforts to implement those above goals from various aspects, especially to bear emission responsibility and take emission reduction actions in response to climate change, as well as to formulate food policy to eliminate hunger and achieve food security. In the field of agriculture, the balance between agricultural carbon emission reduction and product supply guarantee can not only ensure basic needs for human survival, but also help achieve a win-win goal of tackling climate change and economic development, and simultaneously promoting the realization of sustainable agriculture. Therefore, we summarize the existing research on agricultural carbon emissions and agricultural product supply from the following three aspects.

The first is the measurement of agricultural carbon emissions and agricultural product supply. Carbon emissions are often measured from different components, industrial activities and regions, and so are agricultural carbon emissions. Li and Wang (2023) introduced an estimation method of various components about agricultural carbon emissions, and found that China's agricultural carbon emissions presented an inverted “U-shape” from 1995 to 2020 and mainly came from carbon emissions generated by fertilizer utilization [5]. With the deepening of economic globalization, agricultural trade has become an important part of the country's social and economic development. Some scholars not only pay attention to the carbon emissions of agricultural production activities [6], but also attach great importance to the carbon emissions transfer in agricultural trade activities, in which the trade carbon emissions are mainly calculated using the input-output model [7]. From a regional perspective, many scholars pay more attention to spatial patterns. They usually measure agricultural carbon emissions in each region of large areas and compare the spatial differences [8]. Of course, some literature specifically measure agricultural carbon emissions of certain country or region and present some nature and technology of solutions that can enrich carbon emission reduction path, including agricultural carbon sink pathways to provide reference for similar countries or regions to achieve positive spillovers [9]. The research on the supply of agricultural products at present focuses on studying its output, potential, food security, trade barriers and supply chain, while the measurement is less concerned and mainly conducted by unit yield or total output [10,11]. The second is the relationship between agricultural carbon emissions and agricultural product supply. The extreme carbon reduction tendencies such as “mobile carbon reduction” have led to the neglect of agricultural productivity, which attracts attention from all sectors of society. Therefore, some scholars usually use decoupling model and coupling coordination model to demonstrate the relationship between agricultural carbon emissions and grain yield, crop yield and agricultural economic growth, and say that no matter in agriculture or other industries, economic growth and environmental protection should be coordinated to achieve real sustainable development [[12], [13], [14], [15]]. The third is influencing factors. Previous studies suggested that leading officials' accountability audit of natural resources policy [16], agricultural specialization [17], technological progress [18] and others all affect agricultural carbon emissions, whereas the factors affecting the supply of agricultural products include contract farming [19], internet of things [20], environment with technological innovation [21] and so on. Among those, the role of technological progress in agricultural production efficiency and agricultural carbon emission reduction is unquestionable, and becomes the core content of addressing climate change and realizing low-carbon transformation [22]. It follows that under the requirements for high-quality development, the impact of technological innovation is increasing day by day, and regional technology coordination and linkage mechanism cannot be excluded. However, China has an arduous task to achieve the goals of “Carbon Peak” by 2030 and “Carbon Neutrality” by 2060 under imbalanced and insufficient development conditions, if technological progress is “non-clean”, resulting “energy rebound effect” and “non-clean substitution” are not conducive to the sustainable development of agriculture. Green technological innovation is more ecologically representative endogenous driving force for development and itself has the attribute of energy conservation and emission reduction. Compared with the existing research on indistinct and non-targeted agricultural technological progress, green technological innovation is not only conducive to breaking through some technical limitations of “non-clean production”, promoting the realization of ecological products' value, obtaining advantageous pricing power, optimizing industrial structure and other practical issues, but also can help develop and invest agricultural machinery, produce green agricultural products, expand domestic and international markets, and further reduce energy consumption and pollution from the “source” of agricultural production to the “end” of output [[23], [24], [25]]. In other words, fully exploiting the “technological dividend” of green technological innovation is a key to solving the problem of environmental and economic disharmony. In short, climate with the attribute of global public goods has arose wide attention in the world, a series of problems caused by global warming has also become a hot research topic in the academic community, and achieving carbon emission reduction while ensuring the supply of agricultural products is an inevitable choice for further development of agriculture under global warming.

Through the above analysis, it is found that we can improve and deepen from the following aspects: Firstly, with the increasingly complex international situation, the further deepening of opening up and the globalization of addressing climate change, agricultural trade has become one of the important components in China's social and economic development. However, existing research on agricultural carbon emissions, agricultural product supply and their relationship often overlooks trade's role and lacks a comprehensive consideration. Secondly, there are many literature on studying carbon emissions and grain yield from the perspective of narrow-sense agriculture, but they ignore the carbon emissions generated by diesel fishing boats, livestock and poultry breeding, etc, and the demand for agricultural products such as meat, egg and milk is still showing a rigid growth trend. Thirdly, discussing the impact of agricultural mechanization and general technological innovation on agricultural development is hot spots, but rarely from the perspective of green technological innovation, especially its empirical research on carbon reduction effect, growth effect and synergy effect. Therefore, from the perspective of combining trade and broad-sense agriculture, this paper creatively defines agricultural net carbon emissions (ANCE) and evaluates it using the IPCC carbon emission calculation method, environmental input-output model and economic-water-carbon coefficient method, also confines and calculates effective supply of agricultural products (ESAP) from agricultural products' import and export quantity as well as output. Then, based on the measurement results of ANCE and ESAP, histogram, line chart and bivariate spatial auto-correlation analysis are used to demonstrate spatial-temporal characteristics, trade-off-synergy relationship between these two and discuss their current situation. And we integrate green technology innovation, ANCE and ESAP into a unified framework to explore the carbon reduction effect, growth effect and reasonable range of green technology innovation through OLS, FD-GMM, SYS-GMM and SP-GMM methods, and find a path to drive the coordinated promotion between them. Finally, our intention is to provide scientific reference for Chinese decision-makers in the process of promoting sustainable agricultural development under global warming.

## 2 Materials and methods

### 2.1 Measurement of ANCE

#### 2.1.1 Definition of ANCE

In this paper, ANCE is defined as final total agricultural carbon emissions that fully involve and integrate three perspectives: agricultural activities can not achieve zero carbon emissions, carbon emissions can transfer in agricultural trade, and agricultural ecological welfare exists. That is to say, this final result of subtracting agricultural carbon sinks after the sum of agricultural carbon emissions and agricultural trade carbon emissions based on the following text is ANCE on which we study. Moreover, in the process of achieving China's two goals of “Carbon Peak” by 2030 and “Carbon Neutrality” by 2060, it is necessary to clarify the meaning gap between agricultural zero carbon emissions, agricultural net-zero carbon emissions and ANCE mentioned in this paper. Specifically, agricultural zero carbon emissions mean that the total amount of carbon emissions caused by agricultural activities is 0, but agricultural production must be accompanied by carbon emissions, and it is impossible to achieve the so-called agricultural zero emissions. Agricultural net-zero carbon emissions mean that the emission and absorption of carbon in agricultural activities are equal, which is also the essence of achieving “Carbon Neutrality” goal [26]. But it should be noted that the production object of agriculture is living body, carbon emissions such as CH_4_ from paddy rice fields and gut fermentation of ruminants can be reduced but they almost impossibly be net-zero. ANCE refers to ensure that reducible carbon emissions in agricultural machinery and carbon emissions absorbed and transferred by agriculture can offset each other as much as possible. At the same time, the coordinated promotion of agricultural production increase and carbon emissions reduction as well as the necessary carbon emissions for agricultural activities are not ignored, so that agriculture can continue to achieve the “Carbon Neutrality” goal after reaching the “Carbon Peak” goal. Although it is difficult for agriculture to achieve agricultural net-zero carbon emissions, ANCE implies an ideal state: when technology and economy are further developed, the effect of realizing agricultural carbon sinks through natural attributes is gradually improved and the research, development and application of energy-saving, environmentally friendly, green, low-carbon and new technology products have been promoted, which can reduce some inevitable and avoidable carbon emissions of agricultural activities, and then gradually reduce ANCE, finally, carbon control and emission reduction throughout the whole society will make the “Dual Carbon” goal a reality. Actually, from the perspective of specific industries, industry can achieve net-zero emissions through energy substitution, low-carbon technology and others. However, different industrial attributes lead to different carbon emission goals achieved by various industries. Agricultural activities have indeed some immutable natural attributes, blindly reducing carbon emissions means that the supply of agricultural products and food security are threatened. Therefore, studying ANCE has practical significance for China's agriculture to achieve carbon sequestration and emission reduction under the guarantee of food security and the supply of important agricultural products, while providing a reference for a country or region to achieve the “Dual Carbon” goal in the entire industry based on the characteristics of various industries, energy security, industrial chain and supply chain security.

#### 2.1.2 Productive agricultural carbon emissions

There are many studies on agricultural carbon emissions, in which most scholars calculate the total amount or intensity of agricultural carbon emissions from agricultural energy and material input on crop production [27], fishery [28,29] and others, and refer to productive agricultural carbon emissions. The calculation methods and coefficients are referred to the “2006 IPCC Guidelines for National Greenhouse Gas Inventories” [30]. It is worth noting that narrow-sense agriculture mainly refers to planting industry, and its carbon emission sources are generally defined as effective irrigation area, the total sown area of crops, the net amount of agricultural chemical fertilizer, the amount of agricultural diesel, pesticide and agricultural plastic film [31]. While broad-sense agriculture in our study includes agriculture, forestry, animal husbandry and fishery (note: the agriculture mentioned in the following context is the broad-sense agriculture studied in this paper unless otherwise specified), whose carbon emission measurement index system has not been unified at present, with coefficient misuse, carbon sources duplication, unclear sources and other circumstances [5,32,33]. According to our research theme and perspective, this paper improves and tries to perfect the agricultural carbon emission measurement indicator system under a clarification that carbon emissions are not only CO_2_. This indicator system includes four parts: The first is carbon emissions caused by agricultural energy input. We mainly investigate the generated carbon emissions from raw coal, cleaned coal, other washed coal, briquettes, coke, coke oven gas and other gas, crude oil, gasoline, kerosene, diesel, fuel oil, lubricants, bitumen asphalt, petroleum coke, liquefied petroleum gas, refinery gas and other petroleum products, together with natural gas, liquefied natural gas, heat and electricity consumed in agricultural activities. The second is carbon emissions caused by the input of agricultural materials. This part of the indirect and direct agricultural carbon emissions are caused by the loss of soil organic carbon in agricultural tillage, and using fertilizer, pesticide and agricultural plastic film in the process of production. The third is CH_4_ emissions from crop cultivation and NO_2_ emissions from the agricultural land of crops, in which CO_2_ conversion coefficients are 25 and 298 respectively. This kind of carbon emissions directly or indirectly comes from those of five main crops due to a large difference in agricultural planting structure among different regions, such as rice is not major crop in Henan province and other places. Namely, paddy rice (including early, middle and late varieties planted), wheat, corn, soybean and vegetables. The fourth is CH_4_ and NO_2_ emissions caused by livestock and poultry raising. Based on China's actual situation and main types of agricultural products, this paper takes cattle (beef cattle, dairy cow and buffalo), sheep (goat and sheep), pig, mule, horse, donkey and poultry as the research objects to calculate the carbon emissions of this part. Accordingly, above four parts of carbon emissions are mainly generated by agricultural production activities to provide crop products, livestock products and others. So, we call these carbon emissions productive agricultural carbon emissions, which is calculated by formula (1).

(1) $$\text{ACE}=\sum_{i}^{n}e_{i}T_{i}$$

where, ACE is the amount of productive agricultural carbon emissions; i denotes the type of agricultural carbon emission source; e_i_ is the carbon emission coefficient of agricultural carbon source i used, which has been converted by CO_2_ conversion coefficient to ensure that ANCE are finally measured by a unified standard CO_2_ emission (viz. in order to maintain consistency, the unified standard CO_2_ emission is adopted to measure relevant carbon emissions); T_i_ is the amount of agricultural carbon source i used. Above specific coefficients refer to the data in the appendix of the “China Energy Statistical Yearbook” and the “2006 IPCC Guidelines for National Greenhouse Gas Inventories”.

#### 2.1.3 Agricultural trade carbon emissions

With the deepening of economic globalization, the relationship between carbon emissions and international trade is gradually close [[34], [35], [36]]. In agriculture, China is in the position of net import, and its trade activities have guaranteed national food security to a certain extent. However, with the increasingly serious environmental pollution, agricultural trade will be closely linked to our environment, thus agricultural trade carbon emissions must be concerned. Consequently, this paper consults the research by Zhang et al. (2014) [37] to build an environmental input-output model to calculate China's agricultural trade carbon emissions, and conducts five specific steps as follows. Step 1, we assume that there are N provinces (municipalities or regions) in total, and each province (municipality or region) contains R industrial sectors, among which any sector i can be represented by formula (2).

(2) $$x_{i}^{n}=\sum_{j=1}^{N}a_{\text{ij}}^{n}x_{j}^{n}+y_{i}^{n}+e_{i}^{n}-m_{i}^{n}$$

where, x_i_^n^ represents the total output of sector i in province n, a_ij_^n^x_j_^n^ is the intermediate consumption of sector j to sector i, and a_ij_^n^ is the direct consumption coefficient of province n; y_i_^n^ represents the final consumption of sector i in province n, and e_i_^n^ and m_i_^n^ denote the international exports and imports of sector i in province n, respectively. Step 2, the above formula (2) is extended to agricultural sector (viz. that selected and integrated from industry-classified sectors in “China's regional input-output table 2017” and “China energy statistical yearbook”), which is expressed as follows:

(3) $$X^{n}=A^{n}X^{n}+Y^{n}+E^{n}-M^{n}$$

where, X^n^ is the total output vector of province n, and since only trade carbon emissions of agricultural sector are calculated here, the vector or matrix expressed later is a number; A^n^ represents the direct consumption coefficient of province n, Y^n^ represents the final demand of province n, whereas E^n^ and M^n^ respectively represent the agricultural export trade volume and import trade volume of province n. Step 3, we transfer and rewrite the above formula (3) in the following form:

(4) $$X^{n}={\left(I-A^{n}\right)}^{-1}\left(Y^{n}+E^{n}-M^{n}\right)$$

where, (I-A^n^)^−1^ is the Leontief inverse matrix, in which I is the identity matrix. Then, the direct carbon emission coefficient of province n is expressed as d^n^, which is calculated according to the “2006 IPCC Guidelines for National Greenhouse Gas Inventories”. And the process is as follows: the first is to calculate the CO_2_ emission coefficient of each energy, and then calculate the CO_2_ emissions generated by agricultural energy consumption according to formula (1), and finally divide the CO_2_ emissions by the total output value of broad-sense agriculture. Step 4, we change the above formula (4) into the following formula (5):

(5) $$C^{n}=d^{n}X^{n}=d^{n}{\left(I-A^{n}\right)}^{-1}\left(Y^{n}+E^{n}-M^{n}\right)$$

where, d^n^ (I-A^n^)^−1^ represents the complete carbon emission coefficient matrix (1 × 1) of province n. Therefore, Step 5, the calculation formula of agricultural trade carbon emissions in province n is obtained as follows:

(6) $${\text{ATC}}^{n}=d^{n}{\left(I-A^{n}\right)}^{-1}\left(E^{n}-M^{n}\right)$$

where, ATC^n^ is agricultural trade carbon emissions of province n. When ATE^n^>0, it means that province n is in a net export position of agricultural trade carbon emissions. That is, when agricultural trade is carried out, the carbon emissions that should be borne by trade importer are borne by trade exporter province n of China; When ATE^n^<0, it means that province n is in a net import position of agricultural trade carbon emission. In other words, when conducting agricultural trade, trade exporter bears the carbon emissions that should be borne by trade importer province n of China. Attentively, agricultural trade carbon emissions measured in this section, and known as agricultural trade implied carbon emissions as well, refer to the indirect carbon emissions from resource utilization in agricultural trade process (including acquisition, processing and transportation), which are different and do not overlap with the concept of agricultural productive carbon emissions, and then become an important component of ANCE.

#### 2.1.4 Agricultural carbon sinks

While reducing pollution and carbon emission in agriculture, the carbon sink value of agricultural ecosystem should also be taken seriously under China's “Dual Carbon” goal. Agricultural carbon sinks are mainly based on the total amount of CO_2_ absorbed by crops to photosynthesize in the production process, namely agricultural ecological welfare. In this paper, agricultural carbon sinks are measured by calculating these of rice, wheat, corn, bean, tuber, cotton, peanut, rapeseed, sugarcane, vegetable and fruit with wide grown area, high yield and high economic value. The calculation formula is as follows:

(7) $$\text{ACS}=\sum_{i=1}^{m}{\text{ACS}}_{i}=\sum_{i=1}^{m}{\text{CAR}}_{i}\times {\text{AY}}_{i}\times \left(\frac{1-Q_{i}}{H_{i}}\right)$$

where, ACS stands for agricultural carbon sinks, i is a specific crop, whereas m is the total number of crop types (here is 11); CAR_i_ denotes the CO_2_ absorption rate of crop i, AY_i_ is the yield of crop i, and Q_i_ is the water content of fruit when crop i is ripe; H_i_ represents the economic coefficient of crop i, which is the ratio of economic yield to biological yield. The correlation coefficient of crops involved in the calculation of agricultural carbon sinks is cited from the work by Li et al. (2022) [38], Cui et al. (2022) [39] and Chen et al. (2020) [40].

### 2.2 Measurement of ESAP

In China's “agriculture, rural areas and farmers” work, grasping the key of ensuring stable production and guaranteeing supply of grain and important agricultural products is a solid foundation for building an agricultural power, achieving rural revitalization and common prosperity, as well as comprehensively constructing a socialist modern country. However, with the increasingly severe global warming, environmental protection has gradually attracted attention from all walks of life, whether or not agricultural products can be effectively supplied in the process of environmental governance has some uncertainty. Namely, it is still important to implement environmental protection and economic development at the same time, which also provides scientific reference to explore the relationship between ANCE and ESAP. Besides, since the reform and opening up, the output of agricultural products in China has risen steadily, and international agricultural trade is already and will continue to be an indispensable part of the national economic development. And important agricultural products such as soybeans are mainly from the importation, while aquatic products and vegetables are mainly for the export, which also makes China's agricultural supply vulnerable to the challenge of international situation and unstable. Next, we measure ESAP following three steps. First and foremost, based on broad-sense agriculture's category, as well as a few cases in which the production cycle is long, there is no product, and the product output is not easy to be counted in the current year and others, the total output of agricultural products are measured by summing the output of grain, bean, potatoes, cotton, oil, hemp, sugar, sugarcane, sugar beet, tobacco, flue-cured tobacco, vegetable, fruit, tea, meat, milk, wool, cashmere, egg, honey, wood and aquatic products. Then, agricultural product trade is still an important guarantee for ESAP in China, hence the import and export quantity of agricultural products from the first category to the fourth category in 24 chapters of HS code are counted. Last but not least, China's ESAP can be calculated using the total output of agricultural products plus the difference between the import quantity of agricultural products and the export quantity of agricultural products.

### 2.3 Trade-off-synergy relationship analysis

Generally, Pearson parametric correlation test is used for quantitatively expressing the trade-off-synergy between ecosystem services [41]. When Pearson correlation coefficient is greater than 0, it indicates that two of the services have a synergy relationship with increasing or decreasing in the same. When the coefficient is less than 0, it implies that there is a trade-off relationship among the two services. However, although ANCE and ESAP belong to two subsystems of one joint system, ANCE is a negative subsystem. Therefore, if Pearson parametric correlation test is applied, the trade-off or synergy relationship between these two, such as the relationship of one increase and one decrease contains the two opposite situations of the best and the worst, can not be accurately assessed. So, this paper uses the bivariate spatial auto-correlation analysis ((8), (9)) [42,43] to present the trade-off-synergy relationships between ANCE and ESAP. According to the conclusion of Zhang et al. (2022) [31] on the decoupling relationship between agricultural carbon emission reduction and agricultural product supply guarantee, the classification of area can be divided into low-low agglomeration trade-off zone, low-high agglomeration synergy zone, non-significant zone, high-low agglomeration non-trade-off-synergy zone and high-high agglomeration trade-off zone.

(8) $$I_{\text{gkl}}=\frac{n}{\left(n-1\right)\sum_{i}^{n}\sum_{j}^{n}W_{\text{ij}}}\times \frac{\sum_{i}^{n}\sum_{j}^{n}W_{\text{ij}}\left(y_{k}^{i}-\bar{y_{k}}\right)\left(y_{l}^{j}-\bar{y_{l}}\right)}{\sqrt{\sum_{i}^{n}{\left(y_{k}^{i}-\bar{y_{k}}\right)}^{2}}\sqrt{\sum_{j}^{n}{\left(y_{l}^{j}-\bar{y_{l}}\right)}^{2}}}$$

(9) $$I_{\text{lkl}}=\frac{\left(y_{k}^{i}-\bar{y_{k}}\right)}{S_{k}^{2}}\times \sum_{j}^{n}W_{\text{ij}}\left(\frac{\left(y_{l}^{j}-\bar{y_{l}}\right)}{S_{l}^{2}}\right)$$

where, I_gkl_ and I_lkl_ are bivariate global and local Moran's index, respectively; n is the number of spatial units, and W_ij_ is a spatial weight matrix; S_k_^2^ and S_l_^2^ are the variances of attribute values from observed variables k and l; Y_k_^i^ is the attribute value of observed variable k for space unit i, whereas y_l_^j^ is the attribute value of observed variable l for space unit j, and $\bar{y_{k}}$, $\bar{y_{l}}$ are the mean values of attribute values from observed variables k and l, respectively.

### 2.4 Construction of empirical model

In view of the possible nonlinear relationship between green technology innovation and ANCE or ESAP, as well as the lag of feedback time for the above relationships, OLS is first used in this study. On the basis of a clear relationship between explained variable and explanatory variable from OLS regression results, in order to better display time lag effect, solve endogenous problem and introduce spatial factors, we combine Gaussian mixture model to progressively use differential generalized method of moments (FD-GMM), system generalized method of moments (SYS-GMM) and spatial generalized method of moments (SP-GMM) to estimate the effect of green technology innovation on ANCE or ESAP and its heterogeneity impact.

(10) $$Y_{\text{it}}=\beta_{0}+\beta_{1}X_{\text{it}}+\beta_{2}{X_{\text{it}}}^{2}+\beta_{3}Y_{i,t-1}+\alpha {\text{CON}}_{\text{it}}+\mu_{i}+\varepsilon_{\text{it}}$$

(11) $$Y_{\text{it}}=\beta_{0}+\beta_{1}X_{\text{it}}+\beta_{2}{X_{\text{it}}}^{2}+\beta_{3}Y_{i,t-1}+\alpha {\text{CON}}_{\text{it}}+\gamma_{1}W\times X_{\text{it}}+\gamma_{2}W\times {X_{\text{it}}}^{2}+\delta W\times {\text{CON}}_{\text{it}}+\mu_{i}+\varepsilon_{\text{it}}$$

where, i represents a specific province (municipality or region), and t is a specific year; Y is the explained variable, and Y_t-1_ is the time lag term of Y; W is the spatial weight matrix; X is the explanatory variable, X^2^ is the quadratic term of X, and W × X, W × X^2^ is the spatial lag term of X and X^2^ respectively; CON is the control variable, while W × CON is the spatial lag term of CON; β_r_, γ_s_, α and δ are the regression coefficient, in which r can take 1, 2 and 3, while s can only take 1 and 2; μ_i_ is the individual effect, whereas ε_it_ is a random error term. There are four points worth noting. First, because the high requirement for data stability of these empirical models, the dynamic panel data standardized by the Min-Max method are used in the regression estimation and also strongly balanced through the LLC, Fisher and IPS tests. Second, the model expression of OLS, FD-GMM and SYS-GMM is shown in Formula 10, while that of SP-GMM in Formula 11. Third, due to the need to prevent the use of instrumental variables from being unreasonable and the occurrence of heteroscedasticity leading to regression results not applicable, on the basis of determining that FD-GMM and SYS-GMM are effective through sequence correlation test, Arellano-Bond test and sargan test, we also refer to the homovariance theory to deal with the heteroscedasticity problem. Here, the lag term of explained variable is used as the instrumental variable in the FD-GMM for regression estimation, whereas the SYS-GMM estimation method combines the FD-GMM with the horizontal GMM, and we use the lag term of horizontal variable as the instrumental variable of difference equation and the lag term of difference variable as the instrumental variable of original horizontal equation for the estimation. Fourth, if spatial factors are included in this study, we should further optimize the model and use the SP-GMM method to better deal with the endogenous problems in the regression.

### 2.5 Data source and description

Considering the availability and rationality of data, this paper obtains the original data of 30 provinces (municipalities or regions) in China (excluding Tibet, Hong Kong, Macao and Taiwan) from 2006 to 2020 in China Statistical Yearbook, China Energy Statistical Yearbook, China Science and Technology Statistical Yearbook, China Environmental Statistical Yearbook, China Rural Statistical Yearbook, China Agricultural Products Cost and Income Data Collection, the National Bureau of Statistics and the Development Research Center of the State Council Information Network. In terms of data dimension, the reason why we choose the data from 2006 to 2020 for analysis is that China first proposed energy conservation indicator as a constraint during the “11th Five-Year Plan” period, and then putted forward some carbon emission reduction constraint indicators in the “12th Five-Year Plan” and “13th Five-Year Plan” period, indicating that economic development cannot sacrifice environmental protection. In the “14th Five-Year Plan” and the report of the 20th National Congress of the Communist Party of China, it is again emphasized that there is a long way to go to deal with global warming. We believe that the analysis of data during this period can provide reference to continuing to achieve the goals of “Carbon Peak” and “Carbon Neutrality”, as well as mitigating the tensions between resource constraints, environmental pollution and the others. In terms of individual dimension, there are a few scholars who take prefecture-level cities, counties and districts as the research objects to explore carbon emissions. The reason is that some regions mainly focus on the secondary and tertiary industries, makes that agricultural carbon emissions and the output of certain agricultural products are zero or fluctuate abnormally, etc. Therefore, it is more reasonable to use provincial-level data for such analysis. In variable selection, the explained variables are ANCE and ESAP. The explanatory variable is collected by referring to the method of Chen et al. (2022) [44] for green technology innovation. some reasons are as following: green technologies that can be used in agriculture through the China National Intellectual Property Administration are not all within the agricultural patent code, especially the relevant technologies that can be used in all industries; the current green technologies are of mainly universal achievements, the number of green patent applications or authorizations are not divided and included into industries, the definition and measurement methods of green technology innovation are not unified, and agricultural carbon emissions and product supply studied in this paper are extensive. Thus, it is more representative to choose green technology innovation measured by the number of green patent application for research from this perspectives of comprehensiveness, completeness, and comprehensiveness. The control variables include urbanization level, financial support for agriculture, agricultural production conditions, financial environmental protection expenditure, research and development investment and the degree of openness, and are used to compensate for the neglect or abandonment of other factors and related to the explained variable [31,44]. Here, in order to ensure the significance of variable selection and entire study, we conduct a sensitivity analysis based on Lu et al. (2023) [45] and converted the sensitivity to a range of 0–1, in which 0 represents insensitivity whereas 1 representing extreme sensitivity, and the larger the value, the greater the relationship between the variables will be. The results reveal that the sensitivities of green technology innovation to ANCE and ESAP are respectively 0.892 and 0.755, while the average sensitivities of control variables to ANCE and ESAP are respectively 0.562 and 0.886. These indicate that the selected explained variable is responsive to the explanatory and control variables, and the following study can be conducted. The descriptive statistics of non-standard variable data are shown in Table 1.

**Table 1:** Descriptive statistics of the non-standard variables.

Type	Variable	Unit	Measure	Mean	Std. Dev.	Min	Max
Explained variable	Agricultural net carbon emissions (ANCE)	10^4^ tons	See 2.1	3925.674	3292.799	−325.084	18120.670
	Effective supply of agricultural products (ESAP)	10^4^ tons	See 2.2	5312.152	3891.413	174.519	19770.750
Explanatory variable	Green technology innovation (GTI)	pcs	Number of green patent application	5364.256	8968.537	14.000	67258.000
Control variable	Urbanization level (URB)	%	Urban share of total population	0.558	0.137	0.275	0.896
	Financial support for agriculture (FSA)	10^9^ yuan	Local fiscal expenditure on environmental protection	418.440	291.147	22.960	1339.360
	Agricultural production conditions (APC)	pcs	The sum number of rural hydropower stations and rural reservoirs	4542.518	5053.810	0.000	18622.000
	Financial environmental protection expenditure (FEPE)	10^9^ yuan	Local fiscal expenditure on agriculture, forestry and water affairs	116.316	95.704	5.320	747.440
	Research and development investment (R&D)	10^9^ yuan	Product of R&D investment intensity and GDP	383.581	531.767	2.055	3490.160
	Degree of openness (OPE)	%	Ratio of total imports and exports to GDP	0.307	0.345	0.008	1.570

## 3 Results

### 3.1 Measurement results of ANCE and ESAP

#### 3.1.1 Measurement results of ANCE

**Fig. 1:**
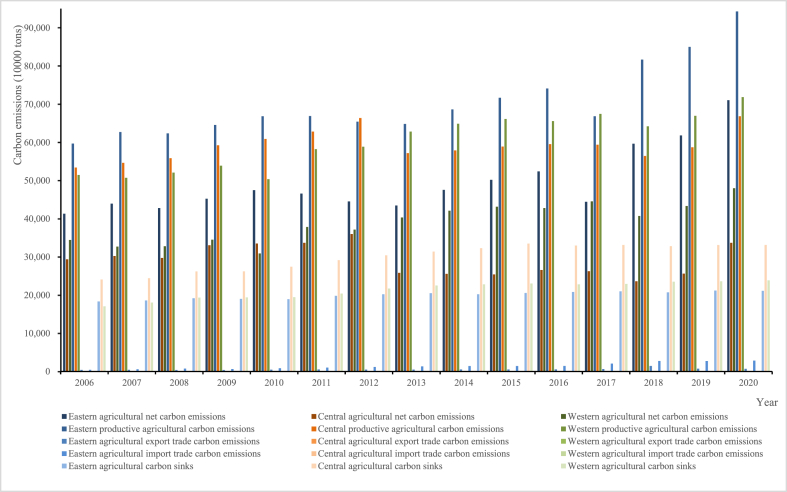
Measurement results of ANCE from 2006 to 2020 in the eastern, central and western regions of China.

**Fig. 2:**
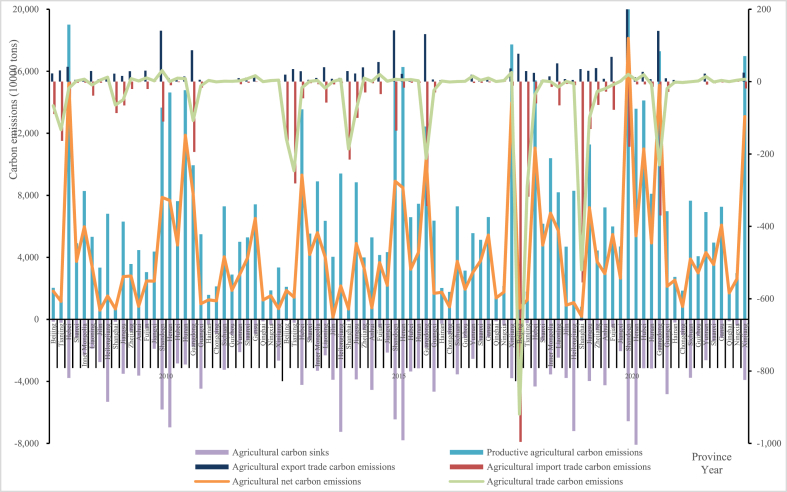
Measurement results of ANCE for each province of China in 2010, 2015 and 2020.

The measurement results of ANCE and ESAP are the basic of the entire study, thus we conduct an in-depth analysis. Before the analysis, we clarify regional divisions of China as follows: Beijing, Tianjin, Hebei, Liaoning, Shanghai, Jiangsu, Zhejiang, Fujian, Shandong, Guangdong and Hainan in the eastern region, whereas Shanxi, Jilin, Heilongjiang, Anhui, Jiangxi, Henan, Hubei, and Hunan in the central region, and Chongqing, Sichuan, Guizhou, Yunnan, Shaanxi, Gansu, Qinghai, Ningxia, Guangxi, Inner Mongolia and Xinjiang in the western region. The measurement results of ANCE in each province of China from 2006 to 2020 are collated and shown in Fig. 1, Fig. 2. As can be seen from Fig. 1, from 2006 to 2020, China's ANCE increased year by year. The main reason for this temporal characteristic is that the needs of social and economic development has to be to met. Agricultural activities have proposed and implemented some solutions based on nature and technology to protect our environment and reduce various types of pollution. But mechanized and large-scale agriculture has taken shape, and some ongoing agricultural carbon emission reduction activities cannot effectively and timely reduce the carbon emissions in agriculture, eventually leading to an upward trend in ANCE. Additionally, ANCE showed a stable increasing trend without significant fluctuations. In fact, this is caused by different characters of carbon emission reduction in agriculture and industry. Some existing and scientific methods mainly address carbon emissions caused by energy consumption in industry, but are limited in the application of agriculture. And necessary carbon emissions for production will steadily increase when agricultural demand raises. Simply put: a series of solutions have been implemented for China's agriculture, but have not yet achieved any obvious effect. In terms of spatial characteristics, ANCE shows a distribution of “higher in the east than in the west than in the center” in China. Specifically, eastern ANCE displays greater up and down fluctuations, western ANCE continues to rise, and ANCE in the central region significantly decreased from 2013 to 2020 compared to those in previous seven years. The reason is not difficult to understand. On the one hand, the policy implementation, technical level, talent status, opening up and other factor in the eastern region are good, and can timely respond to top-down policies and measures. These factors together have made China's eastern region a major agricultural production and trade area, accompanied by a significant amount of carbon emissions. As the leading place of China's agricultural production and trade, the eastern region needs to not only maintain agricultural economic growth, but also become the leading areas for agricultural carbon reduction. The eastern region is a main production area of food and important crops in China, has a large amount of production and investment in fertilizers, pesticides and others, as well as large-scale use of arable land and so on, and produces agricultural production carbon emissions directly higher than those in other regions. Meanwhile, the eastern region is also a major region for agricultural trade. China's agricultural trade is in a unique position of net imports, and so is that in the eastern region. Although some of the country's carbon emissions has been transferred in the eastern regions, but not yet become unfavorable for reducing agricultural carbon emissions. Of course, agricultural activities generate carbon emissions and also have certain carbon sinks that are too small to effectively reduce carbon emissions. Therefore, ANCE in this region accounts for a large proportion of China's totality, and fluctuates very obviously. On the other hand, the both central and western regions have major agricultural provinces of China, but not as good as the eastern region in the natural and social conditions for agricultural development. Moreover, China's central region in the agricultural development conditions are better than the western region, enabling more effective implementation of carbon reduction actions and assuming carbon reduction responsibilities. Then, the spatial-temporal reasons for ANCE can also be traced back to their constituent components. China's productive agriculture carbon emissions have increased year by year, and reached 23,3060.901 (10^4^ tons) in 2020 with the eastern, central, and western regions accounting respectively for 40.465 %, 28.695 % and 30.840 %, which is related to the agricultural structure, scale and support factors in the region. And compared to other types of agricultural carbon emissions in Fig. 1, agricultural trade carbon emissions and agricultural carbon sinks are small but steadily change in a direction that is conducive to reducing ANCE, with the roles greater than those in the central and western regions. One reason is that agricultural imports than exports and agricultural trade itself has low carbon emissions. The other reason is that local agricultural carbon reduction activities based on natural solutions have not yet reached certain scale, and agricultural ecological welfare can only be achieved through cultivating crops. Thus it can be seen, that increasing trend and distribution of ANCE is mainly caused by the increase in productive agriculture carbon emissions, and the role of agricultural trade carbon emissions transfer and agricultural carbon sink' value is though useful but insufficient.

In Fig. 2, we select three end-years (viz. 2010, 2015 and 2020) of the “11th Five Year Plan” period, the “12th Five Year Plan” period and the “13th Five Year Plan” period to analyze China's ANCE, productive agricultural carbon emissions, agricultural trade carbon emissions and agricultural carbon sinks of each province. And if ANCE is obtained by being added to productive agricultural carbon emissions, should be displayed in a positive direction (greater than 0 on the ordinate); otherwise, should be displayed in a negative direction (less than 0 on the ordinate). Due to relatively low carbon emissions from agricultural trade, we build a secondary axis to make it visible. From Fig. 2, the distributions of ANCE in 2010, 2015 and 2020 were similar in poly-line. Beijing, Tianjin, Jilin, Inner Mongolia, Shanghai and Chongqing have relatively low ANCE, while Hebei, Shandong, Henan, Guangdong and Hubei have relatively high one, which is closely related to whether or not they are major agricultural provinces. Among 30 provinces (municipalities or regions) in China, Xinjiang has experienced the largest change in ANCE, with a difference of 1,2388.478 (10^4^ tons) between 2020 and 2010, which is related to China's rigid demand for agricultural products such as grain, meat and egg there. The main grain production areas have been formed in China, but to ensure stable supply of meat, egg and others in the country, the western region, mainly engages in animal husbandry, has kept pace and joined the ranks of ensuring food security and the supply of important agricultural products. In particular, Xinjiang as an important animal husbandry region in China, has a high proportion of characteristic animal husbandry products brought about by special natural geographical and climatic conditions, where the superimposed effect of cattle and sheep raising is gradually emerging. The trend of changes in agricultural productive carbon emissions is basically consistent with the trend of ANCE in China. It is not only an important component of ANCE, but also one of the main sources of carbon emissions in agricultural activities. Agricultural productive carbon emissions mainly show an increasing trend and a significant uneven distribution among provinces, which is related to China's requirements for domestic food security and important agricultural product supply, as well as regional technology, natural resources, and other conditions. At present, the role of agricultural trade cannot be ignored. From 2010 to 2020, the carbon reduction effect brought by agricultural trade was significant and increasing over time. In 2010, 2015 and 2020, the ratios of provinces with agricultural trade carbon emissions less than 0 were 30.000 %, 30.000 % and 66.667 %, respectively. Beijing, Tianjin, Hebei, Liaoning, Shanghai, Jiangsu, Guangdong, Guangxi and Chongqing were net importers of agricultural trade carbon for these three years, whose agricultural import trade carbon emissions have significantly increased and agricultural trade respectively contributed 918.813, 288.718, 35.578, 14.855, 520.526, 100.560, 229.874, 19.085 and 2.1867 (10^4^ tons) to reduce ANCE in 2020. Why? As we all know, over China's large land area, the differences in natural resources, technological levels and others have led to the discrepancies in the agricultural structure of each province. And China's agricultural trade is particularly in a net import position, which indicates that the supply of some agricultural products in the country is insufficient and can only be obtained through imports. The result is that in order to meet the demand for agricultural products and food security of more than 1.4 billion Chinese, local agricultural resources need to be mutually allocated while also introducing and importing agricultural products to make up insufficient domestic supply, ultimately making trade exporters bear the carbon emissions that should be borne by trade importers. Then, we can also find that agricultural carbon sinks in 30 provinces have basically remained stable as shown in Fig. 2, and that productive agricultural carbon emissions with high agricultural carbon sinks are also relatively high, and the trend of change between these two is basically consistent, like in Heilongjiang, Shandong and Henan. It should be noted that Heilongjiang, Shandong and Henan mainly focus on planting, expressing that the availability of agricultural carbon sequestration can be improved through cropping wheat, corn and others to absorb CO_2_ during their growth process. Besides, the difference between productive agricultural carbon emissions and agricultural carbon sinks is not less than 0, which further indicates that agricultural activities need to be carried out, impossibly generate not any carbon emissions at present.

#### 3.1.2 Measurement results of ESAP

**Fig. 3:**
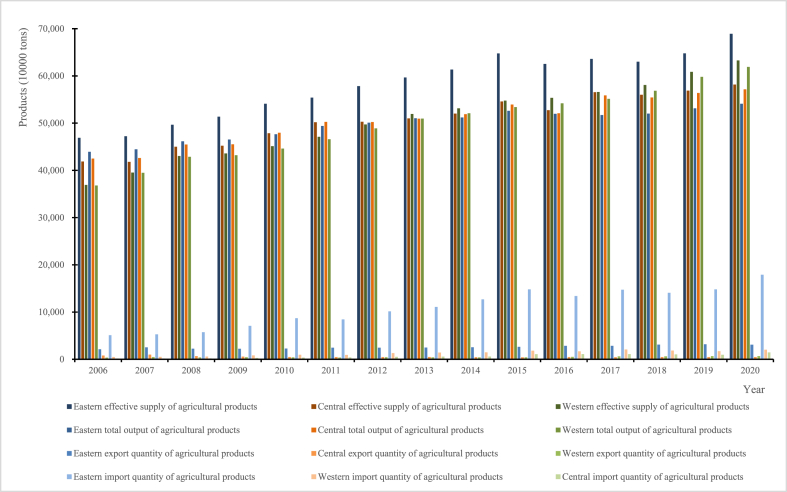
Measurement results of ESAP from 2006 to 2020 in the eastern, central and western regions of China.

**Fig. 4:**
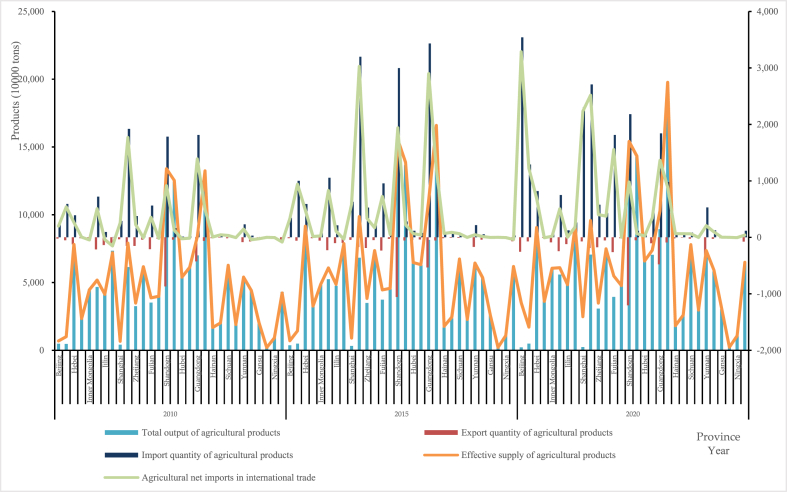
Measurement results of ESAP for each province of China in 2010, 2015 and 2020.

The measurement results of ESAP in each province of China from 2006 to 2020 are collated and shown in Fig. 3, Fig. 4. China's ESAP has increased year by year, and shown spatial distribution in the first seven years as “higher in the east than in the center than in the west” but in the next eight years as “higher in the east than in the west than in the center” in Fig. 3. Reasons accounting for this phenomenon are various. For the increasing trend in ESAP, facing the increasing domestic supply of food and important agricultural products as well as complex international situation, the quantity and quality of agricultural product production in China are increasing. To put it another way, domestically produced soybeans, rice and other products cannot fully meet domestic needs, so a large number of imports are also needed, whereas the main focus is on exporting agricultural products such as fruits, vegetables and seafood that account for a relatively low proportion of China's agricultural products in exports. As a result, the trend of ESAP gradually increases. Notably, ESAP is mainly met by domestic production, supplemented by agricultural trade imports and exports. But improving the production efficiency, product quality, variety and quantity of domestic agricultural products while avoiding vicious trade dependence, is crucial for China facing various traditional and non-traditional risks such as global climate change, trade barriers and green labeling. For the spatial distribution, China's eastern region in ESAP has always been higher than the central and western regions, because the eastern region has unique natural conditions and good social resources such as economy, manpower and technology, and can timely and effectively implement agricultural policies and improve agricultural production efficiency. In 2013, the spatial distribution of “central higher than western regions” in ESAP shifted to “western higher than eastern regions”, for the proposal and implementation of China's “western development strategy” makes the “barren” areas such as Xinjiang in the western region produce more characteristic livestock products and fishery products that overcome technological, natural and other barriers. The combination of these reasons has led to China's ESAP exhibiting the distinctive characteristics mentioned above. Then, the increasing trend and spatial distribution for ESAP is the case with the total output of agricultural products in China. From the perspective of output, the growth trend is caused by the complexity of the international environment and the need for food security, which makes it necessary for China to utilize the domestic market. This means that domestic agricultural production needs to become the dominant force in China's ESAP. And the reason why the eastern region is superior to the central and western regions is that the former not only advanced technology, better natural environment and other conditions for increasing agricultural production, but also a wide variety of agricultural products, especially the variety and total amount of aquatic products being abundant in the coastal areas, and a shift in spatial characteristics from central region to western one is caused by the resources tilting westward. With the deepening of economic globalization, the total number of imports and exports of agricultural products across the country has increased. From 2006 to 2020, the average annual growth rates of China's agricultural product imports and exports were respectively 99.356 % and 62.936 %, whereas those of agricultural product imports in the eastern, central and western regions were respectively 96.528 %, 66.872 % and 68.925 %, and those of agricultural product exports were respectively 63.355 %, −51.196 % and 50.011 %. Moreover, China's ESAP, total output of agricultural products and agricultural product imports reached their peaks in 2015 and 2020, showing a “N-shaped” trend of change. The main information can be obtained as follows from the above. First, the role of agricultural trade in ESAP is gradually increasing, and higher annual growth rate of imports compared to that of exports conforms to current situation in China. Second, the developments of agricultural trade differ between regions. The average annual growth rate of imports and exports in the eastern region is higher than that in the central and western regions, and that of exports in the central region is even negative. Third, agricultural trade has shown a deficit, which can reduce carbon emissions while ensuring food security and the supply of important agricultural products to a certain extent. Fourth, China's overall and sub-regional agricultural trade imports, total agricultural product production and ESAP all reached their tops in 2020. This is the reflection in response to the outbreak of public health and security incidents (viz. Covid-19) started at the end of 2019, and prevent significant challenges and risks from happening to human survival and national security caused by the significant increase in funds used for public health service projects, the emergence of domestic industry stagnation, limited people's production and livelihood, abnormal personnel flow and others. In brief, ESAP in China should be mainly provided domestically, and foreign trade is only a way to add or adjust domestic supply and demand in agriculture. Emphatically, it is crucial to solidly promote China's agricultural development and ensure the supply of domestic agricultural products, as well as a new development pattern dominated by the domestic grand cycle and mutually reinforcing domestic and international double cycles is a major strategic deployment that truly ensures national stability and food security under the world's unprecedented changes in current century and some uncertain and unpredictable international factors.

In Fig. 4, because the imports and exports of agricultural products is relatively small, we establish a secondary axis to help the display clearly. And if ESAP is obtained by subtracting them from the total output of agricultural products, they are displayed in a negative direction (less than 0 on the ordinate), and vice versa. Like ANCE, the distributions of ESAP in 2010, 2015 and 2020 were also similar in poly-line. And ESAP is basically consistent with the quantity and change trend of domestic agricultural products. In these three years, ESAP in Shandong, Henan and Guangxi was higher than that of other provinces (municipalities or regions), and so did the total output of agricultural products. However, except for Guangxi and Xinjiang, where the total output of agricultural products has changed significantly, this part in rest provinces (municipalities or regions) has remained basically unchanged. In other words, each province's ESAP in China has increased year by year and show some difference. So, where does the increase and difference in ESAP come from? The most important factor is the total output of agricultural products. The reason is not only attributed to national increased demand for agricultural product safety and security, but also to the significant differences in the conditions that support ESAP among different provinces. Of course, agricultural trade also plays an important role in ensuring ESAP in China's each province. We can observe that the imports of agricultural products in Fig. 4 was significantly higher in 2015 and 2020 than that in 2010, especially in Jiangsu, Shandong and Guangdong, while that in Shanghai and Beijing was also high in 2020. Among those, Shandong and Guangdong, the two coastal provinces, are the ones that export the most agricultural products with the quantity increase over time, which means that agricultural trade in major agricultural provinces or coastal areas is more active and can effectively rise imports and ensure the stability of ESAP.

Based on the above analysis, reducing ANCE can be achieved through three aspects: reducing productive agricultural carbon emissions, shifting agricultural trade carbon emissions towards import locations and improving agricultural carbon sinks. However, in an increasingly complex international environment, even if agricultural import trade does bring benefits to the supply of agricultural products and carbon emission reduction, the role of trade is still limited. Especially when encountering trade barriers or forming a high degree of trade dependence, the advantages brought by agricultural trade will be transformed into disadvantages, which is not conducive to stable development of national social economy. In addition, the role of agricultural ecological welfare needs to be improved to achieve a reduction in ANCE for China but avoid taking extreme paths, such as not engaging in agricultural activities or planting only crops to help achieve ecological welfare. In short, the task of coordinated development between agricultural carbon emission reduction and product supply guarantee is still a long way to go for China.

### 3.2 Trade-off-synergy relationship between ANCE and ESAP

**Fig. 5:**
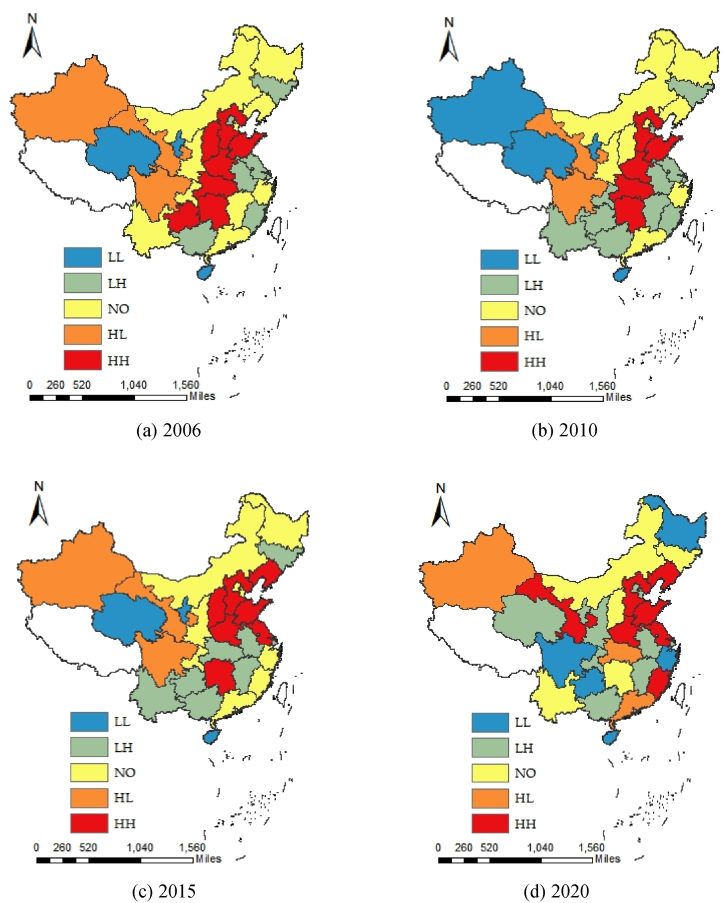
Trade-Off-Synergy relationship between ANCE and ESAP in 2006 (a), 2010 (b), 2015 (c) and 2020 (d) in China.

If agricultural sustainable development is to be achieved, agriculture activity must adapt to current needs, reducing ANCE while ensuring ESAP. The current status of their relationship is an important step to explore their coordinated progress. According to the Pearson parametric correlation test, the relationship between ANCE and ESAP in China was mainly synchronous increase or decrease from 2006 to 2020, and their correlation coefficient range was from 0.350 to 0.520, with the exception of 6 years having negative correlation coefficients between −0.085 and 0.850. As mentioned earlier, ANCE and ESAP are two opposite subsystems of one joint system. Therefore, when the correlation coefficient is less than 0, it can be simply judged that there may be some synergistic relationship or the worst case of non-trade-off-synergy, whereas the correlation coefficient is greater than 0, indicating a trade-off relationship. This means that the relationship between ANCE and ESAP is ambiguous and the applicability of Pearson parametric correlation test is not sufficient. Namely, in this case we cannot determine the specific situation of coordinated promotion between carbon emission reduction and product supply guarantee in China's agriculture. In 2006, 2010, 2015 and 2020, their bivariate global Moran's indices were respectively 0.047, 0.087, −0.071 and −0.158 at a significant level of 5 %, indicating that the relationship between ANCE and ESAP in China as a whole has shifted from a trade-off relationship of equal increase and decrease towards a synergistic relationship of the best or non-trade-off-synergy relationship of the worst. But this result still cannot be used for judging what we need. Therefore, it is need to use the bivariate local spatial auto-correlation analysis for exhibiting the trade-off-synergy relationship between ANCE and ESAP. The results in 2006, 2010, 2015 and 2020 are shown in Fig. 5 (a - d). Notably, the low-low agglomeration trade-off zone (LL) indicates that both ANCE and ESAP are growing at a low speed, which is suitable for the regions dominated by secondary and tertiary industries to make decisions after weighing the advantages and disadvantages in order to achieve the “Dual Carbon” goal in agriculture as soon as possible. The low-high agglomeration synergy zone (LH) is an optimal state for reducing ANCE and promoting ESAP, and acts as important process for achieving coordinated development of economic growth and environmental protection. This state is also applicable to coordinated promotion between ANCE and ESAP in China. The non-significant zone (NO) means no significant relationship between ANCE and ESAP, but does not imply that the potential best or worst relationship between these two will not occur in the future. The high-low agglomeration non-trade-off-synergy zone (HL) shows the worst state, and the situation of high carbon and low yield should be addressed and prevented from rebounding at the current stage. The high-high agglomeration trade-off zone (HH) is the choice of provinces that focus on the primary industry. Currently, ANCE is also necessary for agricultural production. For these provinces that want to ensure national food security and the supply of important agricultural products, it is necessary to maintain national economic development and stability through high carbon and high yield. But zoning the provinces is not always constant, in which it is a common goal for agricultural development now and in the future to shift from a trade-off or non-trade-off-synergy zone towards a synergy one under the conditions permitted by technology, economy, etc. Fig. 5 shows that on the whole, the relationship between ANCE and ESAP in China is gradually getting better, but there are still some provinces (municipalities or regions) with fixed status or repeated advantages and disadvantages. In 2006, among 30 provinces (municipalities or regions) in China, the proportions of provinces (municipalities or regions) that belong to low-low agglomeration trade-off zone, low-high agglomeration synergy zone, non-significant zone, high-low agglomeration non-trade-off-synergy zone, and high-high agglomeration trade-off zone were 10.000 %, 23.333 %, 33.333 %, 10.000 % and 23.333 %, respectively (note: due to the calculated percentage can occur a case of recurring decimal, there may be a possibility that the sum after cycling may not be just 1.000). Then, the proportions of provinces (municipalities or regions) were respectively 13.333 %, 36.667 %, 26.667 %, 6.667 % and 16.667 % in 2010, whereas 10.000 %, 26.667 %, 30.000 %, 10.000 % and 23.333 % in 2015 and 16.667 %, 33.333 %, 16.667 %, 10.000 % and 23.333 % in 2020. Based on these, for temporal characteristics, the number of provinces (municipalities or regions) that belong to low-low agglomeration trade-off zone and low-high agglomeration synergy zone gradually increases, while that belong to non-significant zone gradually decreases, and so do those belong to high-low agglomeration non-trade-off-synergy zone and high-high agglomeration trade-off zone was the same, except for that reaching the lowest proportion in 2010. For spatial characteristics, except for Hebei, Shandong and Henan have always belonged to a high-high agglomeration trade-off zone, Inner Mongolia has always belonged to a non-significant zone, while Shanghai, Anhui and Guangxi have always belonged to a low-high agglomeration synergy zone, Hainan has always belonged to a low-low agglomeration synergy zone, and the rest ones are changing. It can be explained from the following aspects. First of all, after the outbreak of the international financial crisis in 2008, green economy has become an important engine for promoting economic growth, increasing employment opportunities, eradicating poverty and other issues. Since then, most countries in all walks of life have begun to take the path of green transformation, China should and must also keep pace with the world to develop agriculture. Before the “11th Five-Year Plan”, China developed itself with an extensive economy without setting any binding carbon emission reduction targets, which results in severe smog pollution in Anhui, Jiangsu and other provinces, and the country became the world's largest carbon emitter at that time. So, under international emission reduction pressure and domestic harsh environment, in 2010, the number of provinces (municipalities or regions) with high-low agglomeration non-trade-off-synergy zone and high-high agglomeration trade-off zones significantly decreased, whereas those with low-low agglomeration trade-off zones and low-high agglomeration synergy zone increased. Secondly, as a large agricultural country, obviously China can not ignore agricultural productivity in order to reduce carbon emissions, and lead to a situation where Hebei, Shandong and Henan have consistently maintained a high level of net carbon emissions and effective agricultural product supply by decision-making and weighing the pros and cons. But major agricultural provinces in China need to take the lead in reducing carbon emissions while keeping agricultural production. Furthermore, “Green”, “low-carbon” and “sustainable” are important directions for future development. In China, some provinces (municipalities or regions) that can follow the path of low-carbon and high-yield development. For example, Shanghai has always adhered to low-carbon and high-yield development whereas Beijing and Tianjin have been transformed into low-carbon and high-yield development through a series of decisions and measures. And those provinces (municipalities or regions) in an unreasonable position of high carbon and low production need to be gradually transformed. For instance, Gansu has transformed into a high-high agglomeration trade-off zone, whereas Sichuan has transformed into a low-low agglomeration trade-off zone. In general, the implementation of the “Dual Carbon” goal in agriculture in China has already achieved initial results, but the task of ensuring the coordination and unity between agricultural carbon emission reduction and product supply guarantee remains arduous and needs continued efforts. Particularly, for those with currently belonging to the low-low agglomeration trade-off zone, the high-low agglomeration non-trade-off-synergy zone and the high-high agglomeration trade-off zone, it is necessary to continue to implement carbon emission reduction activities and bear the responsibility for carbon emission reduction in agriculture, while paying close attention to ensuring stable production and supply of food and important agricultural products.

### 3.3 Empirical results of ANCE, ESAP and green technology innovation

#### 3.3.1 Regression analysis of OLS, FD-GMM and SYS-GMM

Reducing ANCE while increasing ESAP is a good situation for green technology innovation to achieve the effects of both carbon reduction and production growth. This paper uses OLS, FD-GMM, SYS-GMM and SP-GMM to estimate the impact of green technology innovation on ANCE or ESAP, and thereby identify a reasonable range where green technology innovation can promote the coordinated promotion of ANEC reduction and ESAP guarantee. Although the above bivariate spatial auto-correlation test has been passed, the regression analysis here needs to consider whether there is a spatial correlation for green technology innovation, ANCE or ESAP. Therefore, through spatial auto-correlation testing, we have known that spatial factors could not be considered and SP-GMM was not applicable in this study. That is, to explore the effects of green technology innovation on both carbon reduction and production growth, only OLS, FD-GMM and SYS-GMM are used based on inter-provincial panel data from 2006 to 2020 in China for showing the results in Table 2. Preliminary judges are that the relationship between green technology innovation and ANCE or ESAP is stable, the changes in ANCE and ESAP exhibit “time dependence”, “snowball” effect and “shared weal and woe” effects. And from the regression results of OLS, FD-GMM and SYS-GMM, variables' coefficient direction and significance are basically consistent, in which green technology innovation does have an inverse “U-typed” relationship with ANCE, a positive “U-typed” relationship with ESAP. However, introducing Y_t-1_ and X^2^ often brings about inevitable endogenous problems. Compared with OLS and FD-GMM, the SYS-GMM can not only eliminate the endogenous problems in regression model, but also solve the issue of some missing errors from FD-GMM. Therefore, the regression results of SYS-GMM have more consistence and reasonable empirical performance with theoretical expectations, on which following discussion focuses.

**Table 2:** Regression results of OLS, FD-GMM and SYS-GMM.

Variable	ANCE	ESAP	ANCE	ESAP	ANCE	ESAP
	OLS	OLS	FD-GMM	FD-GMM	SYS-GMM	SYS-GMM
L.ANCE	0.939*** (23.37)		0.482*** (28.26）		0.669*** (31.33)	
L.ESAP		0.791*** (14.04)		0.794*** (31.35)		0.953*** (36.23)
GTI	0.206*** (2.68)	−0.074*** (−3.66)	0.250*** (9.69)	−0.136*** (−18.54)	0.217*** (4.05)	−0.186*** (−19.72)
(GTI)2	−0.114* (−1.78)	0.003 (0.13)	−0.203*** (−9.23)	0.068*** (14.30)	−0.122** (−2.33)	0.100*** (11.58)
URB	0.022 (0.77)	−0.040*** (−2.82)	−0.087*** (−4.25)	−0.029*** (−2.76)	0.227*** (3.21)	−0.042*** (−2.80)
FSA	−0.039* (−1.84)	0.051*** (5.26)	−0.036*** (−4.79)	0.059*** (9.71)	−0.138*** (−8.74)	0.028*** (3.461)
APC	0.010 (0.76)	−0.005 (−0.16)	0.297*** (3.90)	0.022* (1.68)	0.238*** (18.18)	0.020** (2.18)
FEPE	−0.013 (0.24)	0.025 (1.21)	−0.001 (−0.04)	0.022*** (4.69)	−0.233*** (−8.47)	0.046*** (4.78)
R&D	−0.154*** (−2.65)	0.036** (2.70)	−0.127*** (−5.55)	0.041*** (5.81)	−0.058 (−0.74)	0.067*** (11.21)
OPE	−0.012 (−0.05)	−0.028*** (−2.91)	0.097*** (3.64)	0.023*** (4.13)	0.016 (0.59)	0.048*** (4.73)
_cons	0.049 (1.51)	0.069*** (5.34)	0.372*** (14.74)	0.041*** (8.41)	0.164*** (4.57)	0.014* (1.87)

Note: ***, ** and * indicate significant levels of 1 %, 5 % and 10 %, respectively. And they are same in the rest.

When ANCE is taken as the explained variable, the coefficients of GTI and (GTI)^2^ are respectively 0.217 and −0.122 with 5 % significance test passed, showing that green technology innovation has an inverted “U-shaped” impact on ANCE and an inflection point value of 0.889 (about 59,794 pieces). When green technology innovation is below 0.889, it is detrimental to reduce carbon ANCE. Conversely, when green technology innovation exceeds 0.889, carbon reduction effect is reflected. Understandably, in the initial stage of green technology innovation, given that all research, development and utilization conditions supporting green technology innovation are limited, mature green technology products are insufficient and only have fewer similar technologies that can be compared and more expensive than ordinary agricultural technology products, leading to such negative impact of carbon emission reduction through green technology innovation as rising unemployment and increasing costs. Thus agricultural development tends to use traditional and even high energy consumption and pollution agricultural technologies to increase output and achieve economic benefits during this period. However, with the improvement of green technology innovation and other supporting conditions, the function of government has shifted from support to guidance, and the atmosphere for research, development and utilization of green technology innovation across the country has been strengthened. In order to obtain greater benefits and produce green agricultural products that better meet the needs for domestic and international markets, the users can purchase and trust such technology products under mature condition and policy support, thereby shifting the entire agricultural technology progress towards energy conservation and environmental protection, and the carbon reduction effect of green technology innovation can ultimately generate more added value. When ESAP is the explained variable, the coefficients of GTI and (GTI)^2^ are respectively −0.186 and 0.100 at a significant level of 5 %, meaning that green technological innovation has a positive “U-shaped” impact on ESAP with an inflection point value of 0.930 (about 62,551 pieces). When green technology innovation is lower than 0.930, it is mainly designed to protect the environment. And blind use of green technology innovation to improve ESAP is not reasonable and can lead to a decline in ESAP. When green technological innovation surpasses 0.930, scientific research institutions have higher requirements and pursue both environmental protection and economic growth. The characteristics of green technology innovation determine whether its effects on ANCE and ESAP are inconsistent or completely opposite. And it is not that the smaller ANCE or the greater ESAP, the better for China's agricultural development will be, which means finding a reasonable range to achieve the effects of green technology innovation on carbon reduction and production growth at the same time, is crucial. According to Table 2, the reasonable range for green technology innovation in China is greater than 0.930, within which a strong decoupling between ANCE and ESAP can be achieved and synergistically promoted. Nevertheless, through screening the levels of green technology innovation among various provinces, it was found that only Jiangsu and Guangdong in 2019 reached the reasonable range, implying that the level of green technology innovation in China needs to be improved to exert corresponding the effects on carbon reduction and production growth in agriculture. From the above analysis of the relationship between green technology innovation, ANCE and ESAP, we can conclude that achieving the “win-win” of reducing carbon emissions and increasing production, as well as improving farmers' income to promote sustainable growth in agriculture and rural areas is a challenging task in China, but green technology innovation is an effective way. For one thing, with the continuous promotion of ecological civilization construction, green technology innovation will guide traditional agricultural machinery with high energy consumption, high pollution, high cost of research and development, and only play a role in increasing production and income to be replaced by green technology products based on environmental protection. The purpose is that under the same investment in research and development funds, talents and other inputs, obtaining green technology products are more conducive to the high-quality development of agriculture and can achieve a positive interaction between science and technology and agricultural production. And this also means that green technology innovation can replace part of the labor force, achieve large-scale agricultural production, improve agricultural production efficiency, and ensure an increase in the output and quality of agricultural products. For another, global warming has yet not been effectively mitigated, but green technology products brought about by green technology innovation can enable the application and promotion of technologies in agricultural drought resistance, water-saving irrigation and others, improve natural conditions required for the survival of animals and plants, optimize agricultural seed technology, and enhance agricultural production efficiency to address some negative impacts of climate warming on agricultural development. In addition, the coefficients of L. ANCE and L. ESAP are respectively 0.669 and 0.953 at the significance level of 1 %, indicating that both are easily affected by their level at the previous period. In fact, ESAP must be accompanied by the consumption of ANCE. However, unlike their sizes the two coefficients have the same direction, which means that provinces may increase ESAP due to the impact of the previous period, but they will consider environmental issues and reduce ANCE to some extent. Thus it can be seen that all participating entities must quickly and flexibly formulate corresponding measures to prevent agricultural carbon emission reduction from becoming a persistent issue and forming a “rebound effect”, effectively exert the advantage of ensuring ESAP, and simultaneously keep alert and confident in agricultural activities. In terms of control variables, each variable has different effects on the explained variables, which shows that the use of targeted input resources is very critical and important.

#### 3.3.2 Heterogeneity analysis

China has obvious characteristics of imbalanced and insufficient economic and technological development, which makes the significant differences between eastern coastal region and central plus western regions. Therefore, in order to minimize the impact on economic development coordinating environmental protection, China should also pay attention to regional differences in the process of harmonious development between environment and economy, and play the role of green technology innovation in accordance with local conditions. The SYS-GMM regression results of regional heterogeneous impact of green technology innovation on ANCE and ESAP are shown in Table 3. Whether the explained variable is ANCE or ESAP, we can find significant differences in the coefficient significance, size and direction of explanatory and control variables. In the eastern region, there is a significant inverted “U-shaped” relationship between green technology innovation and ANCE, with a inflection point value of 0.553 (about 37,200 pieces). Because the coefficient of (GTI)^2^ does not pass the 10 % significance test, we believe that green technology innovation has a negative impact on ESAP in the eastern region. This indicates that there is no reasonable range in the eastern region to achieve a reduction in ANCE while an increase in ESAP. In the central region, green technology innovation has an “U-shaped” relationship with ANCE and ESAP at a significant level of 10 %, with inflection points of 0.290 (about 19,515 pieces) and 0.186 (about 12,521 pieces), respectively, which means that green technology innovation in the central region focuses on increasing production and puts reducing carbon emission in secondary position in agriculture. In the western region, the impacts of green technology innovation on ANCE and ESAP are respectively an inverted “U-shape” and a positive “U-shape”, with a reasonable range of more than 0.125 (about 8420 pieces), indicating that green technology innovation in the western region focuses on carbon emission reduction and puts increasing production in secondary position in agriculture. The reason for regional heterogeneity of green technology innovation is that under current situation of weak technological innovation capacity and imbalanced regional ability to introduce technology and independently research methods, local green technologies chosen have biases based on their own regional or agricultural development characteristics. For example, economically developed eastern region attaches importance to the overall carbon emission reduction of entire industry home, while central region, as an important grain and rice producing region, pays attention to the supply of agricultural products, and the western region with a complex natural environment and underdeveloped economy, where emphasizes to reduce agricultural carbon emissions. From this point, the improvement of green technology innovation should be not a “bold” unified standard, but a specific case based choice of research and development and use. In other words, targeted development and application of green technology innovation products can create a good external environment for agricultural development from the aspects of optimizing spatial layout structure, improving the market, transportation, agricultural machinery and the like.

**Table 3:** Regression Results of SYS-GMM in the eastern, central and western regions of China.

Variable	Eastern region		Central region		Western region	
	ANCE	ESAP	ANCE	ESAP	ANCE	ESAP
L.ANCE	0.624*** (2.71)		0.657*** (8.81)		−0.474 (−0.45)	
L.ESAP		0.846*** (15.02)		0.779*** (10.6)		1.025*** (88.55)
GTI	0.457*** (2.63)	−0.120** (−2.47)	1.215*** (2.62)	0.176** (2.20)	7.432 (1.44)	−0.325*** (−3.22)
(GTI)^2^	−0.413** (−2.18)	0.023 (0.48)	−2.098* (−1.77)	−0.473* (−1.83)	−73.978* (−1.76)	1.305*** (3.08)
URB	0.322 (1.39)	−0.142*** (−3.55)	0.207 (0.92)	−0.251*** (−3.05)	−1.960* (−1.82)	−0.013 (−0.38)
FSA	−0.341** (−1.98)	0.074* (1.80)	−0.059 (−0.53)	0.102** (2.37)	−3.208 (−1.52)	0.035 (1.56)
APC	0.033 (0.34)	0.035 (1.44)	0.104 (1.44)	−0.043* (−1.91)	−1.665 (−1.51)	−0.004 (−0.09)
FEPE	−0.077 (−1.16)	0.057** (2.17)	0.107 (0.63)	0.087*** (2.74)	9.91 (1.60)	−0.117 (−0.78)
R&D	−0.026 (−0.15)	0.063** (−2.09)	−1.024** (−2.25)	0.006 (0.11)	6.926 (1.26)	0.067 (0.55)
OPE	−0.045 (−0.51)	0.017 (1.11)	1.75 (1.55)	0.252** (2.55)	3.099* (1.70)	0.065** (2.01)
_cons	0.148*** (4.78)	0.094*** (2.96)	0.053 (0.65)	0.115** (2.38)	1.414 (1.49)	0.011 (0.89)

The period of this research is from 2006 to 2020, including the three important periods of “11th Five Year Plan” period, the “12th Five Year Plan” period and the “13th Five Year Plan” period, in which each has respective unique policies and main goals in China. Therefore, in order to more accurately study the relationship among green technology innovation, ANCE and ESAP, we conduct a time heterogeneity analysis of green technology innovation in three time periods: from 2006 to 2010, from 2011 to 2015, and from 2016 to 2020 (viz. The “11th Five Year Plan” period, the “12th Five Year Plan” period and the “13th Five Year Plan” period). The results are shown in Table 4. During the period from 2006 to 2010, green technology innovation has a significant inverted “U-shaped” effect on ANCE with a inflection point value of 0.553, a significant negative impact on ESAP, and without any reasonable range. During the period from 2011 to 2015, green technology innovation has a significant inverted “U-shaped” impact on both ANCE and ESAP, with inflection points of 0.290 and 0.186, respectively. And there is no reasonable range, but a trend towards a reasonable range. During the period from 2016 to 2020, green technology innovation has a significant inverted “U-shaped” impact on ANCE with a inflection point value of 0, a significant positive “U-shaped” impact on ESAP with a inflection point value of 0.125, having a reasonable range greater than 0.125 (about 8420 pieces), and the number of provinces exceeding the value of 0.125 is gradually increasing. According to this conclusion, green technology innovation has significant beneficial effect on reducing ANCE and increasing ESAP, these effects gradually expand over time, and a reasonable range emerged during the “13th Five Year Plan” period that can simultaneously reduce ANCE and increase ESAP. Simply, since China has proposed energy-saving indicators as constraints in the “11th Five Year Plan” period and carbon emission reduction constraints in the “12th and 13th Five Year Plan” period, indicating that economic development cannot abandon environmental protection. This shows that the coordination between environment and economic development has gradually penetrated into various industries, and agricultural production must consider environmental issues to ensure survival needs sustainably. And under a series of supportive conditions, the role of green technology innovation with environmental protection becomes gradually significant and positive. In the “14th Five Year Plan” period and the report of the 20th National Congress of the Communist Party in China, it is once again emphasized that there is a long way to go to address the issue of global warming. Then we boldly anticipate that China's agriculture will use green technology innovation as a crucial and important way to promote the coordinated development of ANCE and ESAP in the future.

**Table 4:** Regression results of SYS-GMM in China from 2006 to 2010, from 2011 to 2015 and from 2016 to 2020.

Variable	from 2006 to 2010		from 2011 to 2015		from 2016 to 2020	
	ANCE	ESAP	ANCE	ESAP	ANCE	ESAP
L.ANCE	1.024*** (21.87)		1.011*** (17.90)		0.110 (1.50)	
L.ESAP		0.960*** (21.45)		0.993*** (81.89)		0.655*** (3.88)
GTI	1.229*** (2.92)	1.236*** (3.54)	−0.392 (−1.23)	0.304** (2.39)	0.335* (1.67)	−0.110*** (−3.21)
(GTI)^2^	−6.685*** (−2.61)	−6.454*** (−3.13)	0.589 (1.23)	−0.308 (−1.37)	−0.196 (−1.15)	0.052** (2.13)
URB	0.063 (0.91)	−0.084 (−1.37)	0.070 (1.09)	−0.056* (−1.83)	1.180*** (4.63)	−0.185 (−1.40)
FSA	−0.225*** (−3.74)	−0.068 (−0.97)	0.076 (1.24)	0.019 (0.78)	−0.558*** (−7.60)	0.067*** (2.80)
APC	0.026 (0.44)	0.096*** (3.16)	0.051*** (2.81)	−0.002 (−0.05)	−0.186 (−0.75)	0.034 (0.91)
FEPE	0.210* (1.85)	0.162*** (2.70)	−0.303*** (−3.21)	0.008 (0.19)	−0.277*** (−2.82)	0.042 (1.61)
R&D	−0.336*** (−2.87)	−0.218** (−2.33)	−0.019 (−0.11)	−0.003 (−0.06)	−0.621*** (−2.91)	0.224*** (3.40)
OPE	−0.030 (−0.58)	0.036 (1.43)	−0.173** (−2.53)	0.058** (2.10)	0.158 (0.74)	−0.124*** (−3.82)
_cons	0.004 (0.10)	0.023 (0.72)	0.016 (0.80)	0.004 (0.25)	0.432*** (3.42)	0.135* (1.79)

As seen earlier, achieving the reduction of ANCE requires for reducing productive agriculture carbon emissions, shifting agricultural trade carbon emissions from exportation to importation, and improving agricultural carbon sinks. Achieving the increase of ESAP requires for increasing total output and net import of agricultural products. However, we know that in an increasingly complex international environment, ensuring national food security and environmental protection cannot be achieved solely by relying on one aspect, which means that the sources of ANCE and ESAP should also be both coordinated and valued. Table 5 shows the differential impact of green technology innovation on productive agricultural carbon emissions (ANCE_1_), agricultural trade carbon emissions (ANCE_2_), agricultural carbon sinks (ANCE_3_), total output of agricultural products (ESAP_1_) and agricultural net imports in international trade (ESAP_2_). Firstly, green technology innovation has positive impact on both productive agricultural carbon emissions and total output of agricultural products, which relates to the maturity of green technology innovation and China's current development situation. Particularly, productive agricultural carbon emissions are mainly emitted to provide agricultural products, so their proportion increases year by year. Secondly, the impact of green technology innovation on agricultural carbon sinks becomes negative because the number of green technology innovation is greater than 0 and the inverted “U-shaped” curve for agricultural carbon sinks crosses 0-point. The reason is that agricultural carbon sinks are mainly affected by crop varieties, planting areas and natural environment. Although, to some extent, green technology can change planting structure, improve environment and others, the role of green technology innovation in this regard is relatively small and limited nationwide, and current application of primary green technology innovation products in increasing agricultural carbon sinks can lead to some adversities. Thirdly, the impact of green technology innovation on agricultural trade carbon emissions and agricultural net imports in international trade is an inverted “U-shape”, with a reasonable range of 0.376–0.759 (about from 25,298 to 51,052 pieces). But fewer and fewer provinces were located in this reasonable range from 2006 to 2020, which indicates that although foreign trade is an important way to transfer carbon emissions or conduct environmental protection, the role of importation is limited, and excessive import is inappropriate. Finally, no matter what kind of above heterogeneity test, the coefficient, direction and magnitude of the lag term, control variables and constant term are different, which shows that when using green technology innovation to promote the coordinated development of ANCE and ESAP, other supporting elements to be invested should also be considered specific circumstances. In summary, green technology innovation should not only be adopted according to local conditions but also be utilized to a limited extent, for in current society, comprehensive consideration into policies, technology, talent and other factors can only truly provide a positive impact on economic development and environmental protection.

**Table 5:** The differential impact of green technology innovation on ANCE_1_, ANCE_2_, ANCE_3_, ESAP_1_ and ESAP_2_ in China.

Variable	ANCE_1_	ANCE_2_	ANCE_3_	ESAP_1_	ESAP_2_
L.ANCE_i_	0.700*** (23.70)	0.981*** (121.24)	0.809*** (146.03)		
L.ESAP_i_				0.674*** (24.99)	0.458*** (19.06)
GTI	0.175*** (4.32)	−0.005 (−0.95)	0.127*** (11.36)	0.068*** (3.25)	0.170*** (15.05)
(GTI)^2^	−0.059 (−1.34)	−0.010* (−1.90)	−0.169*** (−15.25)	0.033 (1.56)	−0.112*** (−15.96)
URB	0.238*** (5.51)	−0.112*** (−13.42)	0.024*** (3.13)	−0.087*** (−4.78)	0.005 (0.39)
FSA	−0.142*** (−7.58)	0.027*** (6.52)	−0.075*** (−20.11)	0.039*** (3.10)	0.168*** (14.21)
APC	0.145*** (3.79)	−0.064*** (−6.49)	−0.048*** (−6.64)	0.063*** (7.46)	0.080*** (11.84)
FEPE	−0.224*** (−16.67)	0.043*** (5.45)	0.183*** (27.9)	0.157*** (9.45)	0.118*** (19.52)
R&D	−0.043 (−0.77)	0.000 (0.06)	−0.034*** (−5.25)	0.114*** (4.46)	0.223*** (18.99)
OPE	0.005 (0.52)	0.030** (2.21)	0.018*** (6.75)	0.082*** (6.23)	0.000 (−0.12)
_cons	0.155*** (5.23)	0.059*** (12.3)	0.034*** (14.17)	0.026*** (4.07)	0.003 (0.65)

## 4 Conclusions

It is the key mission in the future for China to address such outstanding issues as imbalanced, insufficient, uncoordinated and unsustainable development, weak ability to innovate in science and technology, arduous task to protect ecological environment, and difficult work to secure food supply from important agricultural products. Based on such situation, this paper deeply discussed the relationship of ANCE, ESAP and green technology innovation from measurement, trade-off-synergy relationship and influencing factors. The research findings are as follows. Firstly, overall, China's ANCE exhibits a spatial distribution of “higher in the east than in the west than in the center” and a temporal characteristic of increasing year by year; China's ESAP has a increasing trend and the spatial distribution of “higher in the east than in the center than in the west” in 2006–2012 and “higher in the east than in the west than in the center” in 2013–2020. Secondly, average proportions of provinces in 2006, 2010, 2015 and 2020 are 12.500 % belonging to low-low agglomeration trade-off zone, 30.000 % to low-high agglomeration synergy zone, 26.667 % to non-significant zone, 9.167 % to high-low agglomeration non-trade-off-synergy zone and 21.667 % to high-high agglomeration trade-off zone, respectively accounting for China's totality, which indicates that the implementation of national “Dual Carbon” goal in agriculture has already achieved initial results, but still needs to ensure those provinces of low-low agglomeration trade-off, high-low agglomeration non-trade-off-synergy and high-high agglomeration trade-off zones to continue to implement carbon reduction activities and undertake carbon reduction responsibilities, while paying close attention to ensuring stable production and supply of food and important agricultural products. Thirdly, the effects of green technology innovation on production growth and carbon reduction show both inverted “U-shape”, with reasonable range of above 0.930 value. However, the current level of green technology innovation cannot bring about the reduction of ANCE and the improvement of ESAP through “green technology dividend”. Fourthly, green technology innovation not only has significant spatial and temporal heterogeneity impact in China, but also exhibits a differential influence on productive agricultural carbon emissions, agricultural trade carbon emissions, agricultural carbon sinks, total output of agricultural products and agricultural net imports in foreign trade. In short, local governments in China should establish and improve green technology innovation incubation platforms in order to prevent the occurrence of “one size fits all” and “paint big cakes”, and guide all participants to ensure the investment and application of green technology products within a reasonable range of green technology innovation. At the same time, it is required for China to formulate and implement regional differential policies and plan in accordance with local conditions, to develop and apply or transform green technology products in a sensible manner, in order to effectively drive the coordination between the reduction of agricultural net carbon emission and effective supply of major products countrywide.

## Data availability statement

Data will be made available on request.

## Additional information

No additional information is available for this paper.

## CRediT authorship contribution statement

**Lin Zhang:** Writing – original draft, Visualization, Validation, Methodology, Investigation, Funding acquisition, Formal analysis, Data curation, Conceptualization. **Chengzhi Cai:** Writing – review & editing, Supervision.

## Declaration of competing interest

The authors declare no competing interest that could have appeared to influence the work reported in this paper.
